# Salidroside reduces tau hyperphosphorylation via up-regulating GSK-3β phosphorylation in a tau transgenic *Drosophila* model of Alzheimer’s disease

**DOI:** 10.1186/s40035-016-0068-y

**Published:** 2016-11-29

**Authors:** Bei Zhang, Qiongqiong Li, Xingkun Chu, Suya Sun, Shengdi Chen

**Affiliations:** 1 Department of Neurology and Institute of Neurology, Ruijin Hospital, Shanghai Jiao Tong University School of Medicine, 197 Ruijin Er Road, Shanghai, 200025 China; 2 Laboratory of Neurodegenerative Diseases, Institute of Health Sciences, Shanghai Institutes for Biological Sciences (SIBS), Chinese Academy of Sciences (CAS) & Shanghai Jiao Tong University School of Medicine (SJTUSM), Shanghai, 200025 China

**Keywords:** Alzheimer’s disease, Salidroside, *Drosophila*, Glycogen synthase kinase 3β, Tau

## Abstract

**Background:**

Alzheimer’s disease (AD) is an age-related and progressive neurodegenerative disease that causes substantial public health care burdens. Intensive efforts have been made to find effective and safe treatment against AD. Salidroside (Sal) is the main effective component of *Rhodiola rosea L.*, which has several pharmacological activities.

The objective of this study was to investigate the efficacy of Sal in the treatment of AD transgenic *Drosophila* and the associated mechanisms.

**Methods:**

We used tau transgenic *Drosophila* line (TAU) in which tau protein is expressed in the central nervous system and eyes by the Gal4/UAS system. After feeding flies with Sal, the lifespan and locomotor activity were recorded. We further examined the appearance of vacuoles in the mushroom body using immunohistochemistry, and detected the levels of total glycogen synthase kinase 3β (t-GSK-3β), phosphorylated GSK-3β (p-GSK-3β), t-tau and p-tau in the brain by western blot analysis.

**Results:**

Our results showed that the longevity was improved in salidroside-fed *Drosophila* groups as well as the locomotor activity. We also observed less vacuoles in the mushroom body, upregulated level of p-GSK-3β and downregulated p-tau following Sal treatment.

**Conclusion:**

Our data presented the evidence that Sal was capable of reducing the neurodegeneration in tau transgenic *Drosophila* and inhibiting neuronal loss. The neuroprotective effects of Sal were associated with its up-regulation of the p-GSK-3β and down-regulation of the p-tau.

## Background

Alzheimer’s disease (AD) is a progressive and fatal brain disorder, and affects approximately 36 million people worldwide. This number is expected to double during the next 20 years [1]. Neuropathologically, it is characterized by accumulation of extracellular senile plaques consisting of deposits of beta-amyloid (Aβ) and intracellular neurofibrillary tangles consisting of hyperphosphorylated tau protein, which ultimately lead to neuronal loss and brain atrophy [2, 3].

In fact, the tau hypothesis suggests that neurofibrillary tangles in the brain represent a major component of the pathophysiology of Alzheimer’s disease [4], which is attributable to an abnormal phosphorylation of tau protein in the brains of AD patients. Under normal circumstances, tau protein is a neuronal microtubule-associated protein that has a crucial role in assemblage and stabilization of microtubules on neuronal axons and the inhibition of apoptosis [5, 6]. However, when tau is abnormally hyperphosphorylated, it destabilizes microtubules by decreasing the binding affinity of tau, and consequently leads to microtubule destabilization, disruption of the axonal transport system, and ultimately, the formation of intracellular neurofibrillary tangles (NFTs). NFT formation spreads to various brain areas during AD progression, ultimately causing neuronal death [7–13]. Previous studies have shown that increasing tau phosphorylation occurs early in the development of AD [14, 15], and that Aβ associated clinical cognitive decline is identified only following such elevated tau phosphorylation [14, 16]. It is expected that intervening the formation of these toxic assemblies would attenuate the appearance and development of the symptoms of AD. Although many researches have discovered a great deal of pharmaceutical treatments for AD, no effective compound has been found so far for this debilitating neurodegenerative disease.

Over the past decades, drug therapies for AD primarily aim at slowing down the cognitive decline and ameliorating the behavioral symptoms, but the pharmacological effects of these drugs remain unsatisfactory. Salidroside (Sal), as one of the active ingredients extracted from the root of Rhodiola rosea L, which is extensively used in traditional folk medicine in Asian and European countries and has been reported to exhibit various strong pharmacological activities. The main effects of Sal are described as anti-oxidative, anti-apoptosis, anti-inflammatory, anti-cancer, and anti-fatigue effects [17–23]. Additional studies have shown that Sal exerts a neuroprotective effect. For example, Sal is able to protect neurons from apoptosis induced by various factors [24–26]. It remains undemonstrated whether Sal exerts neuroprotection against tau-induced toxicity in AD.

In the present study, we investigated the therapeutic potential of Sal in tau transgenic AD model. We found that Sal treatment could improve locomotor functions and prolong lifespan of AD transgenic *Drosophila*. Moreover, we demonstrated that Sal could protect neurons against tau-induced toxicity, which might be associated with regulation of GSK-3β.

## Methods

### Reagents

Salidroside (Sal, Purity > 99.7%) was obtained from the Green Valley Pharmaceutical Corporation (Shanghai, China). It was dissolved in PBS to a stock concentration of 100 mM and stored at − 20 °C. Donepezil was supplied by Eisai Pharmaceutical Co., Ltd. (Tokyo, Japan).

The following antibodies were used: Phospho-GSK-3β antibody, GSK-3β antibody, Mouse monoclonal Phospho-tau (ser396) antibody and tau (Cell Signaling Technology), Mouse monoclonal anti-β-actin antibody (Sigma–Aldrich, Clone AC-15), HRP-conjugated goat anti-mouse IgG (Jackson Immuno Research Laboratories, PA, USA). All chemicals were purchased from Sigma-Aldrich except those noted otherwise.

### *Drosophila* stocks

All *Drosophila* stocks were maintained at 25 °C under a 12:12 h light: dark cycle at constant 65% humidity as previously described [27]. The flies were raised in 50 ml plastic vials containing standard *Drosophila* medium. Transgenic upstream activating sequence (UAS) carrying human tau was obtained from *Drosophila* Stock Center (Institute of Biochemistry and Cell Biology, Shanghai).

### Longevity assay

New flies were collected within 24 h after eclosion for the experiment. At least 100 flies of each genotype were collected and divided into fresh food vials of 20 flies. Food vials were changed every 2–3 days, and the number of dead flies was counted at that time. The survival times described were given as median standard error of the median. Survival curves were analyzed using Kaplan-Meier estimation and log-rank statistical analysis.

### Climbing assay

Locomotor function of *Drosophila* was measured according to the climbing assay as previously reported [28]. Briefly, 10 male flies per 25 ml tube (*n* = 30 for each group) were placed at the bottom, and given 30 min to recover. After 10 s of climbing, the numbers of *Drosophila* between the 0, 5, 10, 15, 20 and 25 ml scale marks were recorded with a video camera. The experiment was performed three times. The results for each group were calculated by the formula below:

$$ \begin{array}{ll}\mathrm{Climbing}\ \mathrm{Index}\hfill & = \left(\mathrm{flies}\ \mathrm{above}\ 20\ \mathrm{ml}\ \mathrm{s}\mathrm{cale}\ \mathrm{mark}\right) \times 1\hfill \\ {}\hfill & + \left(\mathrm{flies}\ \mathrm{between}\ 15\ \mathrm{and}\ 20\ \mathrm{ml}\ \mathrm{s}\mathrm{cale}\ \mathrm{mark}\mathrm{s}\right) \times 0.8\hfill \\ {}\hfill & + \left(\mathrm{flies}\ \mathrm{between}\ 10\ \mathrm{and}\ 15\ \mathrm{ml}\ \mathrm{s}\mathrm{cale}\ \mathrm{mark}\mathrm{s}\right) \times 0.6\hfill \\ {}\hfill & + \left(\mathrm{flies}\ \mathrm{between}\ 5\ \mathrm{and}\ 10\ \mathrm{ml}\ \mathrm{s}\mathrm{cale}\ \mathrm{mark}\mathrm{s}\right) \times 0.4\hfill \\ {}\hfill & + \left(\mathrm{flies}\ \mathrm{below}\ 5\ \mathrm{ml}\ \mathrm{s}\mathrm{cale}\ \mathrm{mark}\right) \times 0.2.\hfill \end{array} $$

### Histological analysis

For immunostaining analysis, flies (*n* = 10 for each group) were fixed in freshly prepared 4% paraformaldehyde, processed to embed in paraffin blocks, and sectioned at a thickness of 5 μm. Sections were placed on slides, stained with hematoxylin and eosin, and examined by bright field illumination using a Leica DM 2500 microscope at the magnification of 60×. The areas of the vacuoles in the cell body or neuropil regions were captured.

### Western blot analysis

After treatment, fly heads (*n* = 50 for each group) were homogenized in lysis buffer (50 mM Tris–HCl pH 8.0, 150 mM NaCl, 1% NP-40, 0.5% sodium deoxycholate, 0.1% SDS) with protease inhibitor cocktail (Roche, Basle, Switzerland) and 1 mM phenylmethyl sulfonyl-fluoride (PMSF) for 30 min on ice. Total extracts were centrifuged at 14,000 × g for 30 min and boiled in 4× SDS loading buffer for 5 min. The samples were subjected to SDS polyacrylamide gel electrophoresis (SDS-PAGE) and transferred to a polyvinylidene fluoride membrane (Millipore, Bedford, MA, USA). The membranes were blocked using 5% skim milk in TBST for 1 h then incubated at 4 °C overnight with respective primary antibodies to t-GSK-3β(1:1000), p-GSK-3β(1:1000), t-tau (1:1000), p-tau (1:1000) and β-actin (1:5000). After being washed three times with TBST, the membranes were incubated with horseradish peroxidase (HRP)-conjugated goat anti-rabbit/mouse antibody (1:10000) for 2 h at room temperature. Visualized with the indicated antibodies using Immobilon Western Chemiluminescent HRP Substrate (Millipore) and analyzed by ImageJ (National Institutes of Health) software. All the experiments were performed at least three times and the most representative results were shown.

### Statistical analysis

All statistical analysis was performed using SPSS software 19.0(SPSS Inc., Chicago, IL). The Kaplan–Meier test was used to assess the difference in the lifespan curves. Two-group comparisons were analyzed using Student *t*-test. A comparison of three or more groups was performed using one-way ANOVA followed by Tukey’s test. All experiments were carried out in triplicate (*n* = 3) and results were expressed as the mean ± standard error of the mean (SEM). Calculated comparisons were at confidence interval (CI) 95%. A *P*-value < 0.05 was considered statistically significant.

## Results

### Sal prolonged the lifespan of AD transgenic flies

**Fig. 1:**
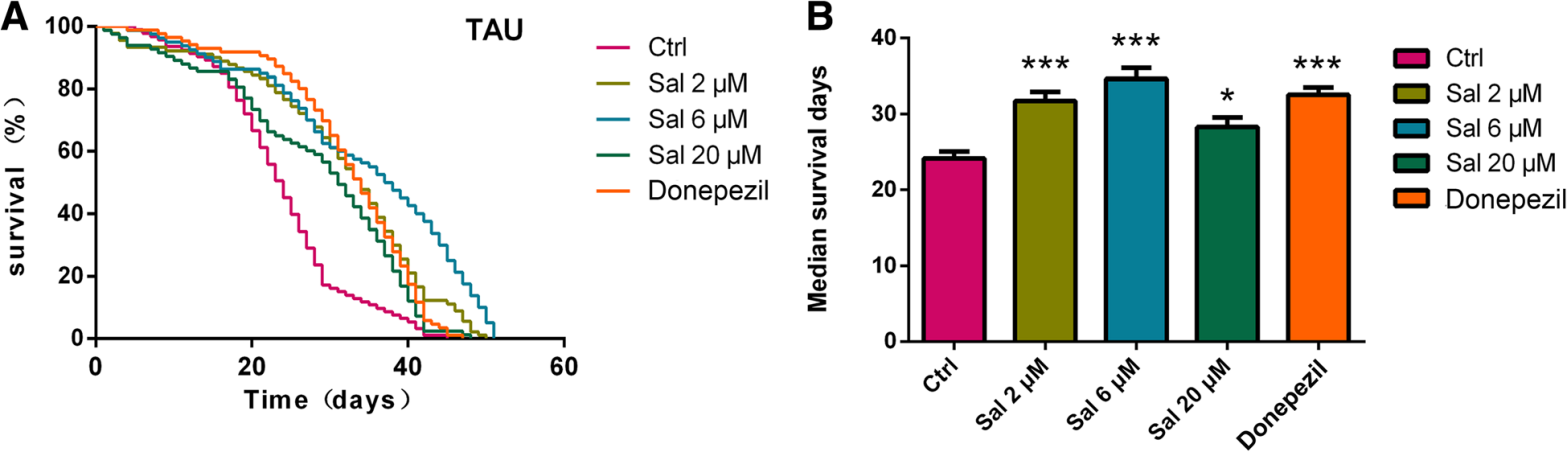
Salidroside treatment improves lifespan of AD transgenic *Drosophila*. **a** Survival trajectories of TAU flies with different treatment. **b** Salidroside treatment prolonged survival time of tau transgenic flies. Donepezil was used as positive Control. Kaplan-Meier cumulative survival analysis was applied to the survival data. Data are presented as mean ± SEM of 3 independent experiments. *P < 0.05, ***P < 0.001

*Drosophila* AD models were generated by expressing human tau, which have been assisted in the identification of novel targets for therapy [29]. These models show intracellular neurofibrillary tangles consisting of hyperphosphorylated tau protein and ultimately significant reduction in longevity [29, 30]. To assess the effect of Sal in living organisms, we firstly fed human tau transgenic flies with Sal in various concentrations (2 μM, 6 μM and 20 μM) or Donepezil (10 μM, the clinically approved drug for the treatment of AD) as positive control and measured their survival duration. We found that the lifespan of Sal-treated flies was more prolonged compared to that of the untreated flies. Sal treatment increased both the survival rate and the median survival time of flies, which is comparable to the improving effect of Donepezil (Fig. 1).

### Sal treatment improved locomotor activity in AD flies

**Fig. 2:**
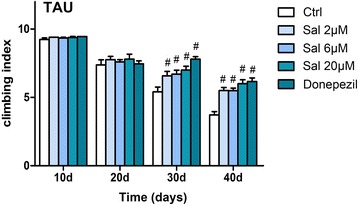
Salidroside increases the locomotor activity. The climbing ability of flies at 10 days, 20 days,30 days and 40 days after eclosion. TAU flies without any treatment showed an activity decrease with increased age but the treatment of Sal and Donepezil enhanced the activity of TAU flies at 30 days or 40 days. The values are mean ± SEM. ^#^ *P* < 0.01 compared to the control group with one-way ANOVA analysis followed by Tukey test

Locomotor assay is a behavioral paradigm to assess the neural functional abnormalities based on the negative geotaxis against gravity. We fed tau flies with Sal or Donepezil at different time points (10, 20, 30, and 40 days), and we found Sal treatment improved the climbing ability of these AD transgenic flies significantly in a dose dependent manner after 30 days compared to the control (the ctrl group) (Fig. 2). However, no obvious difference was observed between the treated and non-treated groups at time points of 10 and 20 days (Fig. 2).

### Effects of Sal on neuronal loss in AD flies

**Fig. 3:**
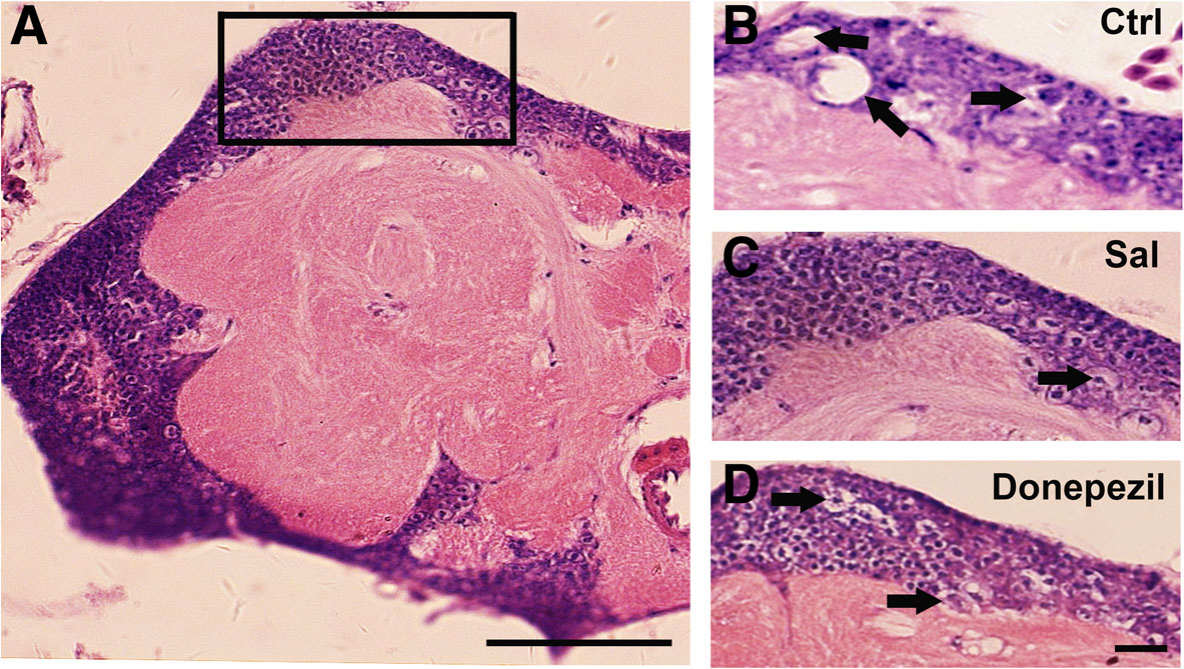
Effect of salidroside on tau-induced neurotoxicity in vivo. Treatment of Sal and Donepezil rescued the neurodegeneration in TAU flies. Hematoxylin and eosin staining of a TAU fly brain (**a**). Hematoxylin and eosin staining of the brain of a TAU fly without any treatment (**b**), TAU fly treated with Sal (**c**), and TAU fly treated with Donepezil (**d**). *Arrowheads* indicate neurodegeneration. Bar:50 μm. Right-panels, Bar: 10 μm

The tau flies were able to replicate the features of human in progressive neurodegeneration with some extent as previously reported [31]. The appearance of vacuoles in the brain is thought as a hallmark of neurodegeneration in *Drosophila*, which represents the neuronal loss of brain [27]. As seen in Fig. 3, the transgenic AD fly model showed numerous vacuoles and exhibited loosely packed neurons all over the mushroom body at postnatal 30 days. Sal treatment in the dose of 6 μM was able to prevent these histological abnormalities in vacuoles and neuronal packing phenotype, which appeared a better therapeutic effect than that in Donepezil- treated group.

### Sal regulated GSK-3β phosphorylation

**Fig. 4:**
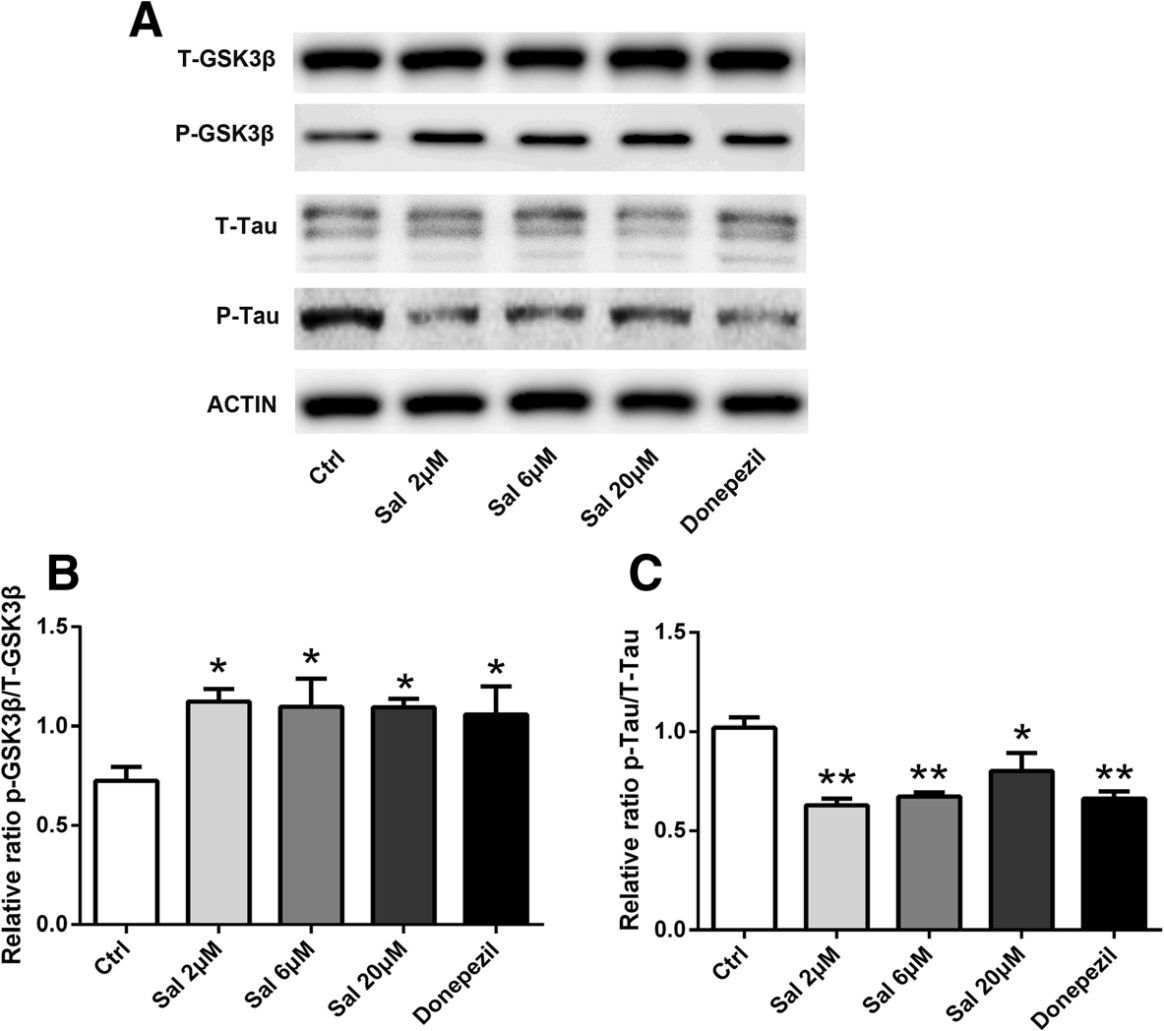
Salidroside inhibits tau-induced neurotoxicity by activating the GSK-3β in vivo. **a** Tau-expressing transgenic flies were treated with Sal or Donepezil for 30 days. The levels of total GSK-3β, total tau, phosphorylated GSK-3β and phosphorylated tau were detected and compared with the control group. **b** The expression levels of GSK-3β, tau and their phosphorylated form were detected. All data are presented as mean ± SEM.*p < 0.05, **p < 0.01 (one-way ANOVA and Tukey’s test)

To explore the signaling pathways that may be involved in Sal effects, we next assessed whether Sal affected GSK-3β phosphorylation and tau phosphorylation in flies, as GSK-3β signal pathway exerts a crucial role in promoting neuronal survival under a variety of circumstances, while tau hyperphosphorylation and microtubule destabilization is widely acknowledged in AD [32–34]. We detected GSK-3β protein expression in *Drosophila* brain after Sal or Donepezil treatment, and found that Sal increased the level of p-GSK-3β effectively while decreased the level of p-tau, a downstream target of GSK-3β (Fig. 4). This result indicates that the neuroprotective effects of Sal in the tau transgenic AD flies might be associated with the regulation of GSK-3β.

## Discussion

During the last decade, *Drosophila* has emerged and been recognized as a powerful model to study human neurodegenerative diseases including AD. Although this model can not detect memory and cognitive function, the short generation time and short lifespan make it particularly amenable to study such age-related disorders [30, 35–37]. In the present study, we showed that Sal treatment prolonged the lifespan and improved locomotor abilities in a tau-expressing transgenic *Drosophila* model. Furthermore, we demonstrated that Sal could dramatically attenuate the neuronal loss in the brains. As far as we know, this is the first evidence for Sal play an important protective role in neurons through up-regulatingGSK-3β phosphorylation in transgenic flies. As Sal was reported with property of non-toxic and mitigated neurotoxicity [38], our study provides a potential promising drug candidate for AD therapy.

In the last two decades, drug discovery and development efforts for AD have been dominated by the “amyloid cascade hypothesis,” focusing on targets defined by this hypothesis and proposing amyloid as the main cause of neural death and dementia. Unfortunately, several clinical trials with anti-Aβ agents failed, thus challenging the hypothesis that Aβ accumulation is the initiating event in the pathological cascade of AD, so we need to explore some novel therapeutic approaches and targets [39]. In recent years, tau-based treatments for AD have become a point of increasing focus and future investigational therapies [40]. Inhibition of the toxicity of tau in the brain may offer significant promise for the treatment of this disease. Our experiments in tau-expressing transgenic *Drosophila* showed that Sal attenuated tau-induced cytotoxicity effectively, suggesting a novel effect of Sal through inhibiting the tau phosphorylation in AD brain.

GSK-3β is a ubiquitously expressed serine/threonine kinase that plays a key role in the pathogenesis of AD. GSK-3β phosphorylates tau in most serine and threonine residues hyperphosphorylated in paired helical filaments [41]. The effect of Sal in the flies increased GSK-3β phosphorylation significantly, while inhibiting tau phosphorylation simultaneously. These results suggest a possible causal relationship for Sal effect between tau hyperphosphorylation and the regulation of GSK-3β phosphorylation. Taken together, the findings of these experiments support the proposition that Sal plays an important role in providing the neuroprotection for AD by regulating tau phosphorylation.

## Conclusion

In summary, we demonstrated that the treatment with Sal relieved the behavioral and pathological changes in a tau transgenic Drosophila model, and the mechanism was associated with its reducing tau hyperphosphorylation via up-regulating GSK-3β phosphorylation. These findings suggest that the Sal may protect neurons from degeneration in brains of AD models, and provide a potential approach in prevention and treatment of AD models. Although Sal has been prescribed to patients with cardiovascular disease and exhibited various pharmacological activities, further multiple studies should be carried out to evaluate the efficacies of Sal against AD.
